# Dynamic Propagation Channel Characterization and Modeling for Human Body Communication

**DOI:** 10.3390/s121217569

**Published:** 2012-12-18

**Authors:** Zedong Nie, Jingjing Ma, Zhicheng Li, Hong Chen, Lei Wang

**Affiliations:** 1 Shenzhen Institutes of Advanced Technology, Chinese Academy of Science, Shenzhen 518055, China; E-Mails: zd.nie@siat.ac.cn (Z.N.); zc.li@siat.ac.cn (Z.L.); hong.chen@siat.ac.cn (H.C.); 2 Shenzhen Key Laboratory for Low-Cost Healthcare, Shenzhen 518055, China; 3 Testing and Technology Center for Industrial Products, Shenzhen 518067, China; E-Mail: jingjing-0816@sohu.com

**Keywords:** human body communication, dynamic channel model, propagation, statistical analysis, motion-insensitive, Fritchman model

## Abstract

This paper presents the first characterization and modeling of dynamic propagation channels for human body communication (HBC). *In-situ* experiments were performed using customized transceivers in an anechoic chamber. Three HBC propagation channels, *i.e.*, from right leg to left leg, from right hand to left hand and from right hand to left leg, were investigated under thirty-three motion scenarios. Snapshots of data (2,800,000) were acquired from five volunteers. Various path gains caused by different locations and movements were quantified and the statistical distributions were estimated. In general, for a given reference threshold è = −10 dB, the maximum average level crossing rate of the HBC was approximately 1.99 Hz, the maximum average fade time was 59.4 ms, and the percentage of bad channel duration time was less than 4.16%. The HBC exhibited a fade depth of −4 dB at 90% complementary cumulative probability. The statistical parameters were observed to be centered for each propagation channel. Subsequently a Fritchman model was implemented to estimate the burst characteristics of the on-body fading. It was concluded that the HBC is motion-insensitive, which is sufficient for reliable communication link during motions, and therefore it has great potential for body sensor/area networks.

## 1. Introduction

With the rapid development of biosensor and wireless communication technologies, body sensor networks (BSN) and body area networks (BAN) are becoming more widely used for healthcare and pervasive applications [1,2]. BSN/BAN systems comprise various wearable or implantable sensor nodes that acquire useful physiological signals and transmit them to a central node, such as a PDA [3]. One major consideration for designing such systems is how to establish reliable, low power and secure wireless communication links [3,4]. Typically, Bluetooth, Zigbee or other Industrial Scientific Medical (ISM) band-based communication protocols are employed. However, the characteristics of the BSN/BAN channel are dynamic due to the motions of the human body, *i.e.*, daily activities such as walking and running, and physiological activities such as the heartbeat and breathing; these activities might affect the wireless propagation channels dramatically. That is why the aforementioned wireless communication technologies are often associated with inevitably deteriorated communication quality [5,6]. It is therefore vital to find a motion-insensitive wireless communication mechanism optimized for BSN/BAN applications.

Human body communication (HBC), which uses the human body as a propagation medium, is a promising solution for body-proximal wireless communications [7–9]. There are several motivations for introducing HBC in BSN/BAN systems. First, compared to the conventional radio frequency (RF) approaches, HBC has a lower power consumption [10]. Second, because the communication distance of a HBC system is constrained around the human body [11], high security and high efficiency in the network utilization can be guaranteed. Third, because an antenna is not required, HBC can be readily integrated within body-worn devices, such as shoes or clothes [8]. Because of the aforementioned merits, the HBC has already been employed in business card [7], touch and play [12] and advertising [13] applications. Recently it was supported by the IEEE standard 802.15.6 for short-range, low-power and highly reliable wireless communication systems for use in close proximity to, or within, the human body [14]. HBC is primarily categorized into two solutions: galvanic coupling and capacitive coupling. The former uses a pair of electrodes in both the transmitter (TX) and receiver (RX) to transmit differential current signals [15–17]. This solution is impractical for BSN/BAN applications whilst the form factor of the complete body-worn devices is extremely miniaturized. The latter uses a single signal electrode for both the RX and the TX, and the ground (GND) electrodes are floating in the air. The signal forward path is established by capacitive coupling with the human body, and the signal return path is formed by coupling with the ambient environment [18,19]. In this way higher data rate and smaller size could be achieved. Therefore, capacitive coupling was investigated in this paper.

Thus far, many researchers have studied the dynamic behavior of various on-body channels. However, most of these studies focused on the on-body channels within ISM bands, such as 868-MHz [20], 2.4-GHz [5,21,22], 2.45-GHz [23–27], 3.1- to 10-GHz [6,28,29], and 4.5-GHz [30,31] bands. Regarding these ISM bands, the channel fading distributions were investigated, and the Lognormal distribution was appeared to be the best fitting distribution [30], a Gamma distribution was found to provide a good fit [5,6], and the level crossing rate and average fading duration were quantified [28]. However, few studies have characterized the dynamic behavior of HBC propagation channels. In HBC, the propagation mechanisms, as well as the carrier frequency bands, are quite different from that of the ISM band communications.

In another side of the spectrum, the majority of published works for HBC have assumed that the body was motionless [15,18,19,32–36], which is not the case for real-world BSN/BAN. References [11,37] reported a preliminary investigation of a dynamic HBC channel, only limited data sets and simple movement scenarios were investigated, therefore conclusions were quite pre-mature.

In this paper, the dynamic HBC propagation channels were characterized. Firstly, the experimental setups with battery-powered transceivers were configured and intensive datasets were acquired. Secondly, the time series of the channel response were introduced, and the channel propagation path gain was statistically tested with given distributions. Thirdly, the statistical parameters such as the level crossing rate and average fade duration were characterized. At last, Fritchman model was presented to describe the burst features, which are the primary characteristics of dynamic changes of the fading channel in human body communication.

## 2. HBC Measurement Configuration

### 2.1. Transceiver

Conventional methods for propagation channel characterizations have employed earth-grounded instruments, such as a sophisticated vector network analyzer or spectrum analyzer. However, such an instrument creates an additional signal return path conducted by the ground [19], and the bulky size makes it difficult to conduct the RF signal measurement during motions, therefore, a battery-powered transceiver was prototyped especially for this study.

**Figure 1.**
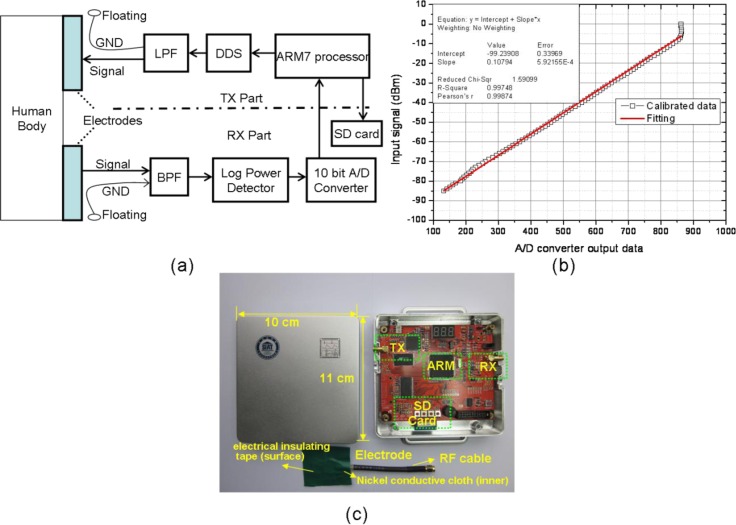
(**a**) Functional block diagram of the measurement system, (**b**) Calibration between the input signal (dBm) and the digital output from the 10-bit A/D converter, (**c**) Top view of transmitting/receiving module with an electrode connected to it.

Figure 1(a) shows the functional block diagram of the measurement system. It included battery-powered transmitter (TX) and receiver (RX) modules. Each TX/RX module was provided with a signal electrode. Electrodes were especially prototyped by us as well. The two signal electrodes were coupled with the human body by capacitive coupling and thus formed the signal forward path (direct electrical contact between the electrodes and the body was not needed). They consisted of nickel conductive cloth encapsulated into plastic electrical insulating tape and connected to the module through a short RF cable. The ground terminals of the two modules were coupled to the surrounding environment by capacitive coupling (*i.e.*, they were freely floating in the air) to form the signal return path [18]. The TX part incorporated a Direct Digital Synthesizer (DDS) in order to provide a 45 MHz sine wave, which falls within the specified frequency band range for HBC [8]; the output sine wave was filtered by a low pass filter (LPF) and then passed to the signal electrode. Similarly, in the RX part, the signal has been gathered by the input electrode and then filtered by a band-pass filter; then the RSSI (Received Signal Strength Indication) of the filtered signal has been estimated by a logarithmic power detector—AD8310 [38,39], the RSSI voltage output of which was sampled by a 10-bit analog-to-digital converter (ADC). The microprocessor stored the measurement data onto a SD-memory card for later off-line analysis. The prototype measurement system was finally calibrated by battery powered signal generator and spectrum analyzer. The measurement system was calibrated and it ensured linear relation between the input signal and the digital output from the 10-bit A/D converter [38,39], as Figure 1(b) shows. By giving a digitalized sample value, the input power could be obtained. Thus, the received power and channel fading were later calculated based on calibration data. It was also observed from Figure 1(b) that the measurement range was from −85 dBm to −6 dBm, and the resolution was approximately 0.1 dBm, which was sufficient for our experiments. Electrode design allowed electrodes to be easily integrated into clothes, shoes, *etc.* In addition, 50 Ù impedance matching was applied in both TX and RX parts [18]. Figure 1(c) shows the transmitter/receiver module with a coupling electrode connected to it. It was assembled into a shielded box, suitable for on-body HBC measurements.

### 2.2. Measurement and Scenario Configurations

**Figure 2.**
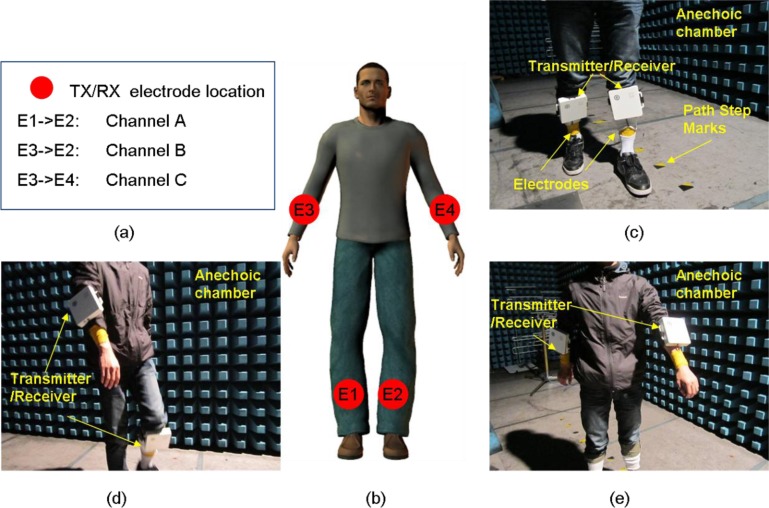
Measurement design. (**a**) Channel configuration and TX/RX locations. For example, ‘E1->E2: Channel A’ indicates that channel A was defined as the channel between locations E1 and E2. (**b**) Electrode locations. E1 was located on the right lower leg, E2 was placed on the left lower leg, E3 was located on the right wrist, and E4 was placed on the left wrist. (**c**) Photograph of the channel A measurement scenario. (**d**) Photograph of the channel B measurement scenario. (**e**) Photograph of the channel C measurement scenario.

To accurately measure the fading caused by movements, all experiments were performed in an anechoic chamber for which the multipath fading from surrounding objects was negligible. Since different physical characteristics may affect the propagation channels [19,40], it is necessary to measure a large amount of data for different people and different movement configurations. In our study, five volunteers (average age of 25 years) with body weights of 50 to 80 kg and body heights of 165 to 180 cm were selected. Written informed consents were obtained from all volunteers. The on-body movement scenarios were configured according to the IEEE 802.15 TG6 proposal, which provides references for usage models [41]. Three HBC propagation paths, as shown in Figure 2, were investigated. We have studied the channel reciprocity by shifting the locations of the transmitter and the receiver. It was found that there was no significant difference after the shifting of the locations. For all of the experiments, the electrodes were fastened with Nexcare bandages, as shown in Figure 2(c).

The path gains of channels A, B and C were investigated *in-situ* for different body movement scenarios. For channel A, the volunteers were asked to walk in a straight line according to specific step lengths and intervals. Path step markers located on the floor and ticking sound played via a loudspeaker were used to calibrate the steps. Step lengths of 20 cm, 40 cm and 60 cm and step intervals of 600 ms, 800 ms, 1,000 ms, 1,200 ms and 1,500 ms were selected. For channel B, volunteers were asked to walk on the spot and wave their hands at an acute angle between the torso and the hand(s) at intervals of 600 ms, 800 ms, 1,000 ms, 1,200 ms and 1,500 ms. Channel C was investigated in two ways. In the first investigation (termed channel C1), the volunteers were asked to wave their hands in the same direction, whereas in the second investigation (noted as channel C2), they were required to wave their hands in opposite directions. The hand waving intervals were set to 600 ms, 800 ms, 1,000 ms, 1,200 ms and 1,500 ms. For comparison, a static characterization of the three channels without body movement was also conducted.

Each scenario was measured with a sampling rate of 100 Hz. A 100 Hz sample rate was sufficient, because it was verified that the frequency of various body movements was always less than 20 Hz [42]. The number of sampling events for each scenario is listed in Table 1. In total, 360,000 to 1,600,000 data samples were collected for each channel and approximately 2,800,000 snapshots of channel responses were captured from the five volunteers. Compared with the previous study, our data sets were capable of supporting intensive evaluations of the dynamic channel fading during routine daily activities [20,25,30,31].

## 3. Channel Characterization

### 3.1. Mean and Standard Deviation of the Path Gain

The mean and standard deviation (STD) of the path gains (PGs) for each scenario are listed in Table 1. The mean path gain showed little variation for different movement scenarios for the same channel, and the STDs were all less than 5.5 dB. Channel A demonstrated the highest path gain, whereas channel C had the smallest standard deviation. In channels A and B, the mean path gain for motionless volunteers (Scenario 1 and Scenario 17) was higher than that for volunteers in motion. For channel C, the motionless mean path gain (scenario 23) was lower than the path gain in channel C1 but higher than that in channel C2. We also observed that as the step length increases, the mean path gain in channel A also increases.

### 3.2. Time-Dependent Characteristics

**Figure 3.**
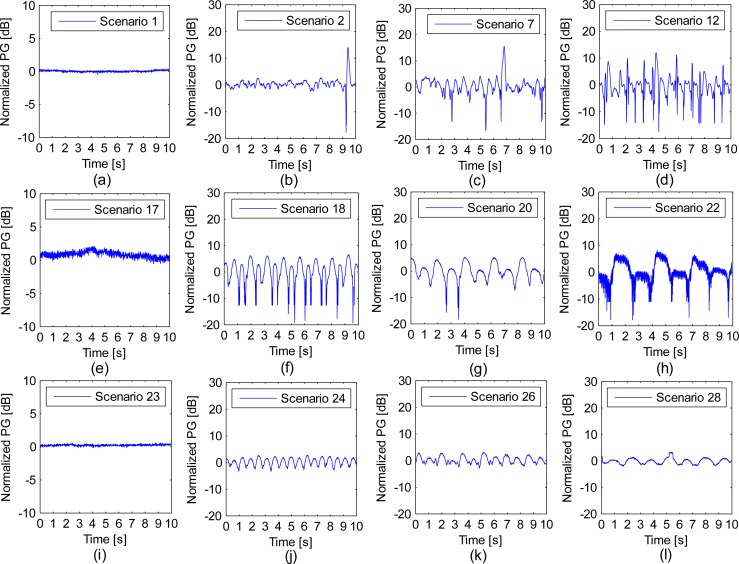
Temporal variations in the normalized path gain across 10 seconds of snapshot data for (**a**) scenario 1, (**b**) scenario 2, (**c**) scenario 7, (**d**) scenario 12, (**e**) scenario 17, (**f**) scenario 18, (**g**) scenario 20, (**h**) scenario 22, (**i**) scenario 23, (**j**) scenario 24, (**k**) scenario 26 and (**l**) scenario 28.

Figure 3 depicts the exemplary time series of the normalized path gains for different path lengths and moving intervals. In order to study the impact of different path lengths, scenarios 1, 2, 7, 12 were selected. In order to find the impact of different moving intervals, scenarios 17, 18, 20, 22 and scenarios 23, 24, 26, 28 were selected. Here, all of the measured path gains were normalized to the mean value of each scenario so that we could focus on the dynamic properties of the propagation channels. The zero value of the y-axis indicates the reference mean path gains, and the normalized value of the snapshot data may be less than or greater than the zero value, as shown in Figure 3. It is interesting to observe that the temporal variation follows a regular baseline along with the repetitive actions. Figure 3(a,e,i) illustrates the normalized path gain measured when the volunteer stood still in scenarios 1, 17 and 23. Fluctuating values were observed in these scenarios. Figure 3(b–d) shows the time-dependent characteristics of different walking step lengths (20 cm, 40 cm and 60 cm) with fixed time intervals (600 ms); when we used a larger step length, the fading depth and fading times both increased, which is clearly shown in Figure 3(b–d). Figure 3(f–h) presents the temporal variations of the different movement intervals for channel B: the fading times are greater in Figure 3(f) than in Figure 3(g,h), whereas Figure 3(f) shows a fast movement rhythm (600 ms). Figure 3(i–l) depicts the time-series of different movement intervals for channel C1, the behaviors of fading were the same as those of channel B. In addition, it was found that the fading depth of channel C was lower than that of channels A and B. This is shown in Figure 3.

### 3.3. Statistical Distribution Analysis

To capture the dynamic characteristics of the HBC, the first-order statistics of the normalized path gain were examined. Seven commonly used probability density functions (PDFs), *i.e.*, Lognormal, Gamma, Weibull, Rician, Normal, Rayleigh and Nakagami distributions were adopted. All distribution parameters in each channel were obtained using the maximum likelihood estimation (MLE), and the best distribution model was then selected based on the Akaike information criterion (AIC), which has been discussed in detail elsewhere [43].

Table 1 summarizes the best-fit distribution models. Gamma distribution was found to be the best fitting model in channel A (best fit in 10 cases), while the other best-fitting cases were all Lognormal. For channel B, Gamma distribution performed well in scenarios of motionless, 1,000-ms, 1,200-ms and 1,500-ms moving intervals. Lognormal and Weibull provided the best fit in scenarios of 600-ms and 800-ms moving intervals, respectively. Interestingly, the Lognormal distribution best described channel C in all cases.

To determine whether alternative distributions existed, the ΔAIC value based on the difference between the AIC for the best-fit and next-best models were calculated (Table 1). As suggested in [44], alternative models may exist when the AIC difference ΔAIC ≤ 10. Based on the obtained ΔAIC values, we determined that the Nakagami distribution could serve as an alternative model for scenario 17 because of the ΔAIC = 9, and no alternative models existed in the other scenarios.

**Figure 4.**
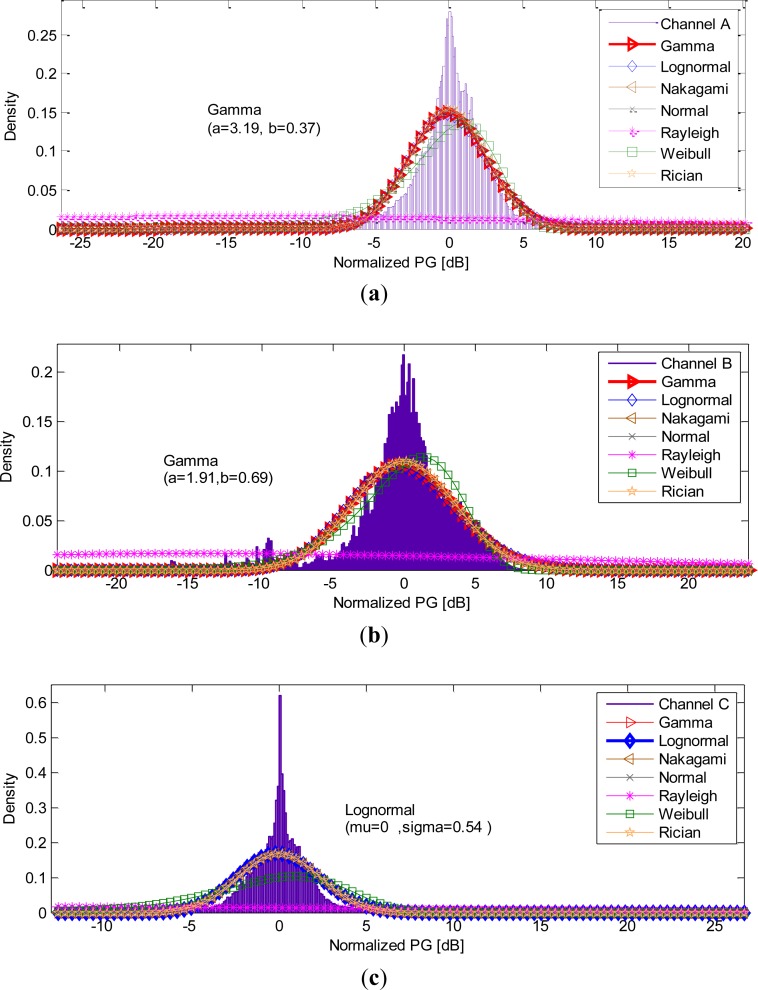
PDFs and distribution fits for (**a**) channel A, (**b**) channel B and (**c**) channel C.

In order to get a general concept model that is independent of movement scenarios, we combined all the measurement data in each channel. Plots of the PDFs and the distribution fittings of channels A, channel B and C are presented in Figure 4.

The bin size for the histogram used to describe each PDF based on the measured data was chosen according to the “Freedman–Diaconis” rule [44]. Figure 4 shows that the Rayleigh distribution performed poorly in all configurations. In addition, the Lognormal, Nakagami, Gamma, Rician and Normal distributions, were approximately coincident. The best fit and the estimated parameters for each channel are also presented in Figure 4. The Gamma distribution showed the best fit for channels A and B, whereas the Lognormal was found to best characterize channel C.

### 3.4. Level Crossing Rate and Fade Duration

The level crossing rate (LCR) and average fading duration (AFD) are the two most important statistical parameters in wireless communication and can be used to select the most suitable error-protection coding scheme and interleaving algorithm in channel modeling [24].

**Figure 5.**
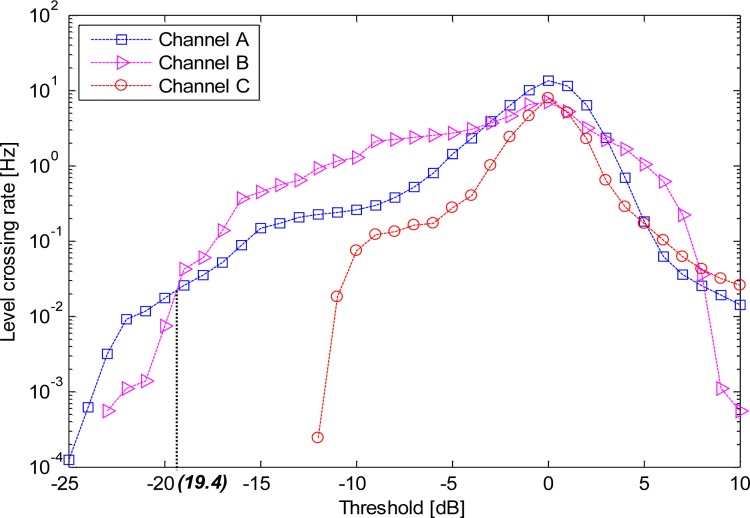
LCRs for the different channels.

Figure 5 shows the average level crossing rate (LCR), which is the frequency at which a signal crosses a threshold in a positive direction in channels A, channel B, and channel C. A correlation between the LCRs and the channels was observed. Notably, channel C presented the lowest LCR. Based on our experiments, whilst the threshold was less than −19.4 dB, Channel A achieved a higher level crossing rate (LCR) than channel B; whilst the threshold was greater than −19.4 dB, the LCR of channel A was lower than that of channel B. This was clearly observed from Figure 5. Given a threshold è = −10 dB, the average LCRs for each scenario are listed in Table 1. All the values of LCR were found to be less than 2 Hz, and in channel C2, the values of LCR were 0 Hz. Here, è = −10 dB was selected as it is usually adopted as the maximum link margin in propagation analysis [26,29].

**Figure 6.**
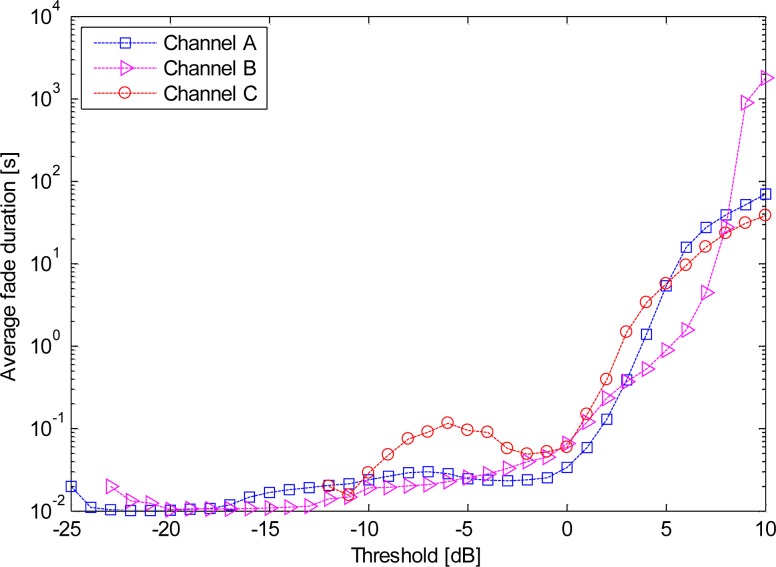
AFDs for the different channels.

The average fading duration is determined as the ratio between the total time that the received signal remains below a reference threshold è, and the total number of fading events. This ratio helps determine the most likely number of bits that may be lost due to fading. Figure 6 shows the AFD for different channels. The AFD values for the threshold è = −10 dB are also shown in Table 1. The maximum of AFD was approximately 60 ms in scenario 5, whereas channel C2 had no fading. Interestingly, compared to the dynamic on-body channel investigated at 2.45 GHz [25], the HBC presented here achieved lower AFD values.

**Figure 7.**
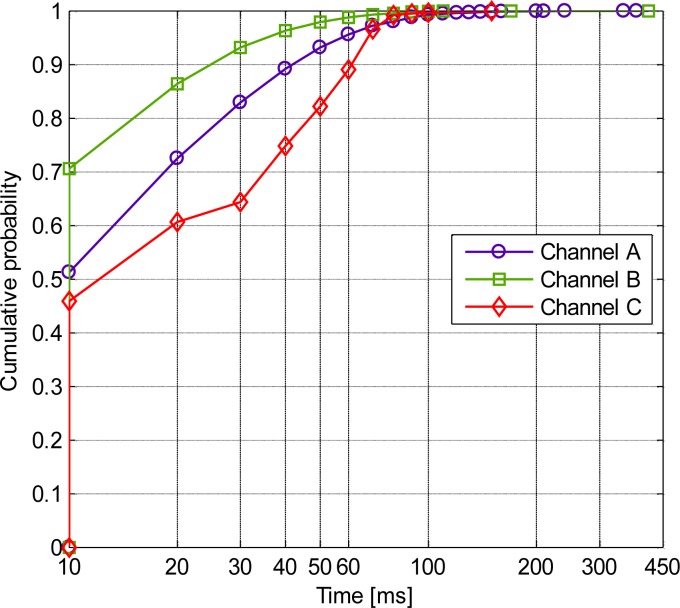
CDF for the bad channel fade duration.

The cumulative distribution functions (CDFs) of the fading duration behavior investigated at è = −10 dB are presented in Figure 7. The CDF of a bad channel fading duration time, which is the total time that the channel path gain was lower than the threshold è, is shown in Figure 7(a). The channel was considered bad when the path gain dropped below the threshold è; otherwise, the channel was considered good. We observed that the fading duration was dependent on the channels. In addition, the majority of the fading duration (bad channel; CDF of approximately 0.9) for channel A was less than 40 ms and for channel B less than 30 ms. Moreover, none of the fading durations exceeded 450 ms. Furthermore, the percentage of the fading duration or the percentage of the time when the channel was bad is summarized in Table 1. All the moving scenarios of channel C2 were all good channels, whereas the worst channel occurred in channel B within the 800-ms moving interval.

In order to evaluate the dispersions of the aforementioned statistical parameters for each channel caused by different movements, two statistical dispersion methods, *i.e.*, range and standard deviation were adopted. The evaluation results are presented in Table 2.

The range parameter demonstrated the differences between the maximum and minimum values of the analyzed data. From Table 2, the maximum values of range for level crossing rate, average fade duration and percentage of the bad channel time were 1.97, 0.059, and 4.14, respectively. Moreover, the standard deviations for channel A, channel B and channel C in those three analyzed parameters were all kept in lower and stable levels.

**Figure 8.**
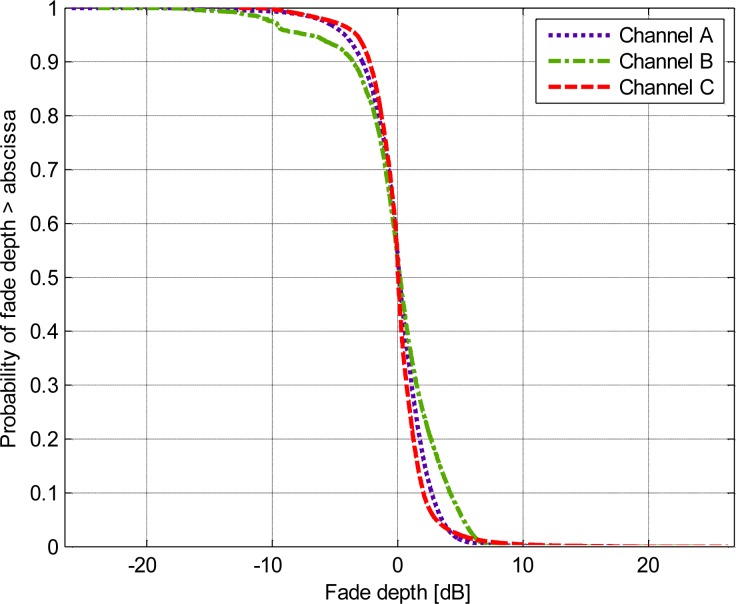
Complementary cumulative distribution functions of the fade depth.

Figure 8 presents an investigation of the fade depth. The worst performance for this parameter was demonstrated by channel B, whereas the majority of the fade depth was greater than −4 dB (ordinate value of 0.9), and 10% of the fade depth had a positive value greater than 5 dB (ordinate value of 0.1).

### 3.5. Discussion

The temporal characteristics shown in Figure 3 revealed that the path gain of the HBC propagation channel varied with time. Even in the motionless scenarios (scenarios 1, 17 and 23), involuntary movements (such as the heartbeat and breathing) may cause path fading fluctuations.

Table 1 shows that the path gains were location-dependent, which can be explained by the coupling mechanism of the HBC. In HBC, the forward signal path was primarily coupled through the human body, whereas the signal return path was coupled with the ambient environment (such as the air and the ground) [18,45]. As the distances of signal forward paths were observed to be almost identical in channel A, B and C, the signal return path had a great influence on the mean path gain. Channel A had the shortest signal return path in distance, which was established between the two lower legs via air, therefore channel A demonstrated the highest path gain.

The signal return path varied and played important part in the HBC dynamic propagation channels, because it changed along with the body motion, while the signal forward path underwent minor change as it was coupled with the body and the locations of the RX and TX were fixed. For example, in channel A, the main signal return path was established by air, mainly from the right lower leg to the left lower leg, walking scenarios conducted longer air path, resulting in lower path gain than motionless scenario. Inversely, the movement in channel C1 decreased the signal leakage from the human body, conducting higher path gains. For channel C, the impedance of signal return path was relatively high and underwent minor changes during the motion, due to the long distance between the two wrists, therefore, the smallest standard deviation and the lowest level crossing rate were observed in channel C. Body movement also caused the signal return paths to vary in time with the same rhythm. For example, Figure 3 shows that the temporal variations have regular patterns synchronized with the specific movement scenarios. A longer step length meant a longer distance between the transmitter and the receiver. The prolonged distance caused a longer signal return path; therefore, both the fade depth and fading time were increased. This is clearly shown in Figure 3(b–d). To summarize, the variations of the signal forward path and the signal return path in distance only resulted limited variations of fading depth and fading times, therefore, lower values of statistical parameters (*i.e.*, AFD) were observed.

The distributions of the path gains were channel-dependent. The Gamma and Lognormal distributions provided the best fits, which are similar to that of the ISM bands. Moreover, the Rayleigh distribution performed poorly in all configurations. It suggested that HBC has a dominant component representing the signal coupling with the body and the ambient environment; movement has no significant influence on the propagation channel. In addition, the assumption of no obvious multipath effect in HBC was suggested in literatures [11,18,46]. Therefore, we adopted the conventional analytical methods to model the HBC channel fading.

As we have discussed before, the propagation fading due to the body motion makes the communication link in ISM bands unreliable; moreover, the body motion is usually irregular and unpredictable, which causes difficulties to precisely compensate the channel. Fortunately, due to the limited variation in the coupling path of the HBC channel during the body motion, we observed several interesting results when we applied further detailed statistical analysis to the HBC channel: (1) Based on the observations made for Figure 5 to Figure 8, it can be concluded that statistical parameters were all dependent on the channels. (2) The statistical parameters for HBC were centered for each propagation channel by performing statistical dispersion analysis. Moreover, the AFD and the bad channel percentage were both achieved lower values. (3) The value of the fade depth was only −4 dB when the complementary cumulative probability was at 0.9. Therefore, it can be concluded that the HBC is channel-dependent. However, the findings in (2) and (3), as well as the little variation mean path and low standard deviation for each channel, depicted that HBC is motion-insensitive.

In BSN/BAN applications, the sensor nodes are usually located in fixed positions according to their applications, for example, a pulse oximeter is usually placed on the fingertip or earlobe. Although HBC suffers from the channel dependent effect, the performance of HBC could be guaranteed by pre-calibration. Most importantly, the motion-insensitive characteristic of the HBC propagation channel could provide a reliable communication link independent of various motions; it is suitable for BSN/BAN applications, such as the real-time of dynamic physiological signal detection and body motion-state monitoring. Furthermore, the lower AFD and bad channel percentage guarantee the high quality of the HBC channel link, therefore the complexity of consequent CODEC and medium access control (MAC) protocol could be simplified. The low fade depth relieved the RF front-end design, thus a low-powered, small-volume RF transceiver design was possible.

## 4. Channel Modeling

Based on the experiments, a three-state Fritchman model was proposed to describe the burst feature of the HBC channels. The finite-state Markov model was studied by Gilbert [47] and Elliott [48], and has been successfully used to characterize fading channels in wireless communication systems [49,50]. For time-variable, flat-fading channels, the finite-state Markov model of the received signal amplitude or received signal-to-noise ratio (SNR) has been frequently studied since 1995 [51]. The Fritchman model, which divides the state space into k good states and N-k bad states, appears to be suitable for modeling burst errors in mobile radio channels [52]. The good states represent error-free transmissions, and the bad states are those that consistently produce a transmission error.

**Figure 9.**
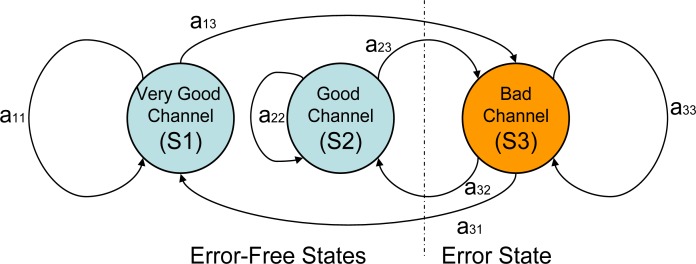
The three-state Fritchman model for dynamic HBC channels.

According to the time series and statistical analysis of the measurement data in the aforementioned sections, the fade depth of the path gain reaches various thresholds. The concept that the received signal strength is above an acceptable performance threshold for part of the time and is below the threshold during a deep fade represents one of the most important criteria for evaluating the quality of a propagation link. Therefore, we modeled the channel to be in one of the three states shown in Figure 9.

In our investigation, we combined all of the measurement data in scenarios 1 to 33, to obtain a general conceptual model that is independent of the channels and movement scenarios. As shown in Figure 8, the thresholds è1 = −4 dB and è2 = 5 dB were selected, which was a reasonable partition in many practical FSMCs (finite-state Markov channels), where the worst and the best channels only occupied a small part within the whole channel. Assuming the sampled signal amplitude keeps the same level during each sample period, the three Fritchman states were defined as follows:

(1) $$\text{States}=\{\begin{array}{ll} S1:\;\text{Very}\;\text{good}\;\text{channel},\;\text{error-free} & \text{PG}>\theta 2\\ S2:\;\text{Good}\;\text{channel},\;\text{error-free}, & \theta 1\leq \text{PG}\leq \theta 2\\ S3:\;\text{Bad}\;\text{channel},\;\text{error}, & \text{PG}<\theta 1 \end{array}$$

The state transition matrix is:

$$A=\left[\begin{array}{ccc} a_{11} & 0 & a_{13}\\ 0 & a_{22} & a_{23}\\ a_{31} & a_{32} & a_{33} \end{array}\right]$$

Upon observation of an error, the state that produced the error cannot be identified when a transition occurs between the two good channels; thus, a zero transition probability between S1 and S2 was set. The error generation matrix takes a very simple form:

(2) $$B=\left[\begin{array}{lll} 1 & 1 & 0\\ 0 & 0 & 1 \end{array}\right]$$

The measured data were classified into different states, and the Baum-Welch algorithm was used to calculate the transition matrix [53]. To reduce the computing time required to test the convergence of the iterative estimation process, the initial state probability vectors **Π_0_** were predefined based on the probability of the fade depth, which is displayed in Figure 8, and the initial state transition matrix A_0_ was rapidly computed based on the measured data. The initial state probability vectors, the initial state transition matrix and the estimated transition matrix A are listed in Table 3.

Based on the estimated Fritchman model, we observed that the transition probability from S1 to S3 was only 0.0062, whereas the transition probability from S2 to S3 was approximately 0.5, which indicates that the HBC channel demonstrated a relatively slow-burst behavior. This model provides the mathematical guidance for HBC system design, for example, the model could be adopted to predict the communication link quality and choose a suitable retransmission strategy in the MAC layer design.

## 5. Conclusions

This paper presents *in-situ* characterization of the HBC dynamic propagation channel in an anechoic RF chamber and investigates the statistical model of the HBC. The contribution and originality are summarized as follows: (1) to the best of our knowledge, we are the first to demonstrate that the HBC fading channel is motion-insensitive by means of intensive *in vivo* experiments, *i.e.*, with five subjects, 33 scenarios and 2,800,000 snapshots of data. A battery-powered ‘floating ground’ transceiver system was implemented particularly for these experiments. We draw this conclusion because we found that: (a) the mean path gain undergoes minor change during different motions within a specific channel; (b) the level crossing rate, the average fade duration, and the standard deviations of the path gain exhibited relatively small values during motions. (2) to the best of our knowledge, we are the first to propose a Fritchman model to describe the dynamic channel fading of the HBC.

In the near future we will investigate the subtle variations of the channel fading under different environments, such as laboratory, grove, playground, conference hall, and with extended durations (*i.e.*, from days to weeks).

## Figures and Tables

**Table 1.** Measurement design and statistical analysis of the measurement data.

**Scenario**	**Channel**	**Step length (cm) #**	**Moving interval (ms)**	**No. of samples**	**Mean (path gain) (dB)**	**STD (path gain) (dB)**	Best fit *	**ΔAIC**	Level crossing rate (Hz) **	Average fade duration (s) **	Percentage of bad channel time (%) **
1	Ch. A	0	0	100,000	−45.7	2.0	L	1575	0.16	0.0236	0.37
2		20	600	100,000	−49.1	4.0	L	6573	0.07	0.0432	0.30
3		20	800	100,000	−48.7	4.2	G	9873	0.06	0.0400	0.24
4		20	1000	100,000	−48.5	4.2	L	2196	0.01	0.0408	0.04
5		20	1200	100,000	−48.4	4.0	G	861	0.02	0.0594	0.09
6		20	1500	100,000	−48.1	4.0	L	3862	0.55	0.0241	1.31
7		40	600	100,000	−49.9	5.0	L	8030	0.47	0.0228	1.07
8		40	800	100,000	−49.6	5.1	G	5681	0.20	0.0265	0.52
9		40	1000	100,000	−49.3	4.6	G	1671	0.11	0.0312	0.33
10		40	1200	100,000	−49.2	4.5	G	7962	0.18	0.0273	0.47
11		40	1500	100,000	−49.4	4.6	G	1566	0.94	0.0177	1.69
12		60	600	100,000	−50.4	4.9	G	6632	0.61	0.0185	1.12
13		60	800	100,000	−50.2	4.9	G	4692	0.35	0.0236	0.81
14		60	1000	100,000	−50.1	4.7	G	13230	0.22	0.0224	0.48
15		60	1200	100,000	−50.1	4.4	G	10621	0.20	0.0211	0.42
16		60	1500	100,000	−50.1	4.4	L	31985	0	0	0

17	Ch. B	-	0	60,000	−55.8	0.94	G/N	9	0.02	0.0107	0.02
18		-	600	60,000	−56.1	5.13	L	1634	1.71	0.0152	2.59
19		-	800	60,000	−55.9	5.13	W	559	1.99	0.0209	4.16
20		-	1000	60,000	−55.9	5..0	G	43	1.08	0.0177	1.90
21		-	1200	60,000	−55.9	4.7	G	821	1.12	0.0119	1.33
22		-	1500	60,000	−56.6	4.7	G	520	1.79	0.0126	2.25

23	Ch.C1		0	140,000	−52.3	1.6	L	10474	0	0	0
24		-	600	40,000	−49.8	5.2	L	19148	0.59	0.0329	1.93
25		-	800	40,000	−47.9	4.6	L	11411	0.43	0.0284	1.21
26		-	1000	40,000	−48.6	4.3	L	12138	0.49	0.0253	1.24
27		-	1200	40,000	−50.0	3.5	L	1238	0.01	0.0200	0.01
28		-	1500	40,000	−50.6	3.1	L	694	0	0	0

29	Ch.C2	-	600	100,000	−53.5	1.9	L	19918	0	0	0
30		-	800	100,000	−53.7	2.5	L	8750	0	0	0
31		-	1000	100,000	−53.1	2.8	L	14440	0	0	0
32		-	1200	100,000	−53.1	2.2	L	1843	0	0	0
33		-	1500	100,000	−53.6	1.7	L	671	0	0	0

#: ‘−’: Volunteer was asked to walk on the spot.

*: L: Lognormal, G: Gamma, N: Nakagami, W: Weibull.

**: Threshold è = −10 dB.

**Table 2.** Results of the statistical dispersion of level crossing rate, average fade duration and percentage of bad channel time for channel A, channel B and channel C.

	**Level crossing rate (Hz)**		**Average fade duration (s)**		**Percentage of bad channel time (%)**	

	**Range**	**STD**	**Range**	**STD**	**Range**	**STD**
Channel A	0.94	0.26	0.059	0.013	1.69	0.48
Channel B	1.97	0.72	0.010	0.003	4.14	1.37
Channel C	0.59	0.24	0.032	0.013	1.93	0.71

**Table 3.** Three-state Fritchman model for a dynamic HBC channel.

**Initial Π_0_**	**Initial A_0_**	**Estimated A**
$\Pi_{0}=\left[\begin{array}{ccc} 0.1 & 0.8 & 0.1 \end{array}\right]$	$A=\left[\begin{array}{rrr} 0.8 & 0 & 0.2\\ 0 & 0.9 & 0.1\\ 0.0002 & 0.3447 & 0.6551 \end{array}\right]$	$A=\left[\begin{array}{rrr} 0.9938 & 0 & 0.0062\\ 0 & 0.5341 & 0.4659\\ 0.1064 & 0.2420 & 0.6517 \end{array}\right]$
